# Hierarchical meso/macro-porous carbon fabricated from dual MgO templates for direct electron transfer enzymatic electrodes

**DOI:** 10.1038/srep45147

**Published:** 2017-03-23

**Authors:** Hiroto Funabashi, Satoshi Takeuchi, Seiya Tsujimura

**Affiliations:** 1 Division of Materials Science, Faculty of Pure and Applied Sciences, University of Tsukuba, 1-1-1 Tennodai, Tsukuba, Ibaraki 305-8573, Japan

## Abstract

We designed a three-dimensional (3D) hierarchical pore structure to improve the current production efficiency and stability of direct electron transfer-type biocathodes. The 3D hierarchical electrode structure was fabricated using a MgO-templated porous carbon framework produced from two MgO templates with sizes of 40 and 150 nm. The results revealed that the optimal pore composition for a bilirubin oxidase-catalysed oxygen reduction cathode was a mixture of 33% macropores and 67% mesopores (MgOC_33_). The macropores improve mass transfer inside the carbon material, and the mesopores improve the electron transfer efficiency of the enzyme by surrounding the enzyme with carbon.

Enzymes are more environmentally and economically friendly than precious metal-based catalysts because enzyme-catalysed chemical reactions proceed under ambient conditions and exhibit high substrate and reaction selectivity. However, the industrial use of enzymes is limited by their low stability and restricted operating conditions, requiring moderate temperatures and aqueous solvents to function. The immobilisation of enzymes on a solid support, including sol-gel silica, polymer beads, and glasses, may overcome these drawbacks and facilitate their continuous or repeated function in chemical processes^1^. Especially, porous supports with high specific surface areas have been studied because the amount of immobilised enzyme can be increased. As per IUPAC conventions, porous material can be categorized into three types: macroporous (pore diameter >50 nm), mesoporous (2–50 nm), and microporous (<2 nm). Micropores are so small that they cannot encapsulate enzyme, whose average diameter is in the range 4–20 nm. Thus, macroporous materials have been employed as enzyme support^1^. Mesoporous materials with narrow pore size distributions, which should be tailored to the target enzymes, and high specific surface area have been investigated as enzyme supports to increase the total amount of active enzyme on the support^2^,^3^. Enzymes can be stabilised by encapsulating them in the pores of a support; this prevents the removal of enzymes from the support surface, their aggregation, or degradation of their three-dimensional (3D) molecular structure. The enzyme-support interactions can be affected by the pore characteristics, including the pore structure and morphology, and by surface chemical characteristics, such as hydrophobic/hydrophilic interactions, electrostatic interactions, and hydrogen bonding. Among these factors, the pore size is an important parameter affecting enzyme immobilisation. For example, Takahashi *et al*. reported that the stability of enzymes increased after their immobilisation on ordered mesoporous silica, which was synthesised using surfactants as a template^4^. They also reported that this stabilisation depended on the mesopore size; the enzyme was stabilised by encapsulating it in mesopores with pore sizes comparable to that of the enzyme. Since the discovery of ordered mesoporous silica, this material has been widely studied for the immobilisation of enzymes such as hydrolases, and the effects of pore size, pore structure, morphology, and surface properties of immobilisation of the enzyme have been determined.

Efficient heterogeneous electron transfer is critical in the field of electro-enzymatic reactions. The electrode reactions can be categorised as involving either mediated or direct electron transfer based on the electron transfer route between the enzyme active site and the electrode. Most redox enzymes require redox mediators to accomplish electron transfer between the active site and electrode because the active site of the enzyme is deeply buried in the insulative protein shell (mediated electron transfer [MET] reaction). In contrast, the electroactive sites of some enzymes are near the enzyme surface. These enzymes can exhibit electrocatalytic activity without redox mediators via direct electron transfer (DET) reactions and can be used for the fabrication of continuous operating sensors and biofuel cell (BFC) devices without the requirement to consider the leaching of redox mediators from the electrode surface. However, a drawback of DET systems is their low current output, which is limited by the number of electroactive enzymes on the electrode surface. This limiting factor is determined not only by the molecular size of the enzyme but also by the enzyme’s orientation; the electron transfer rate between the enzyme and electrode depends on the electron transfer distance. Thus, most of these enzymes show no electrocatalytic activity if the orientation of the enzyme is not controlled, such as on a flat electrode, because of the long electron transfer distance. Silica-based porous enzyme supports are not electrically conductive; thus, enzymatic and electrochemical reactions on porous silica electrodes must be connected by redox mediators, including NAD^+^ (NADH), H_2_O_2_, and other redox-active molecules^2^,^3^. The diffusion of electrons (via redox mediators) between the silica support and the electrode decreases the current production efficiency.

Mesoporous electro-conductive materials, including metals, metal oxides, and carbons, can increase the efficiency of the enzyme by reducing the electron diffusion distance for electrons in both MET and DET reaction systems. Rational synthesis of mesoporous metals has garnered considerable attentions^5^. Although mesoporous metals, e.g. Au and Pd, have high electronic conductivities and appropriate potential windows for bioelectrocatalysis, they are more expensive and less biocompatible than carbon materials. In contrast, sp^2^-hybridised carbon-based porous materials have high electric conductivity and are chemically and physically stable compared with silica-based porous materials^6^. The selection of a suitable mesoporous carbon may increase the stability and current production efficiency of redox enzymes, making them suitable for use in enzymatic BFCs to convert biochemical energy into electricity, and for biosensors to monitor continuously bio-related substances, such as blood sugar, under ambient conditions with high selectivity and sensitivity.

The strategies used to synthesise mesoporous carbons for enzyme immobilisation can be categorised into several types. The first strategy involves the use of interstices of carbon particles, such as carbon gels (carbon aerogel and carbon cryogel). The material can have tuneable and narrow pore size distribution. Tsujimura *et al*. reported efficient electro-enzymatic reactions using carbon gels as electrode materials for several multicopper oxidases, including laccase, bilirubin oxidase (BOD)^7^, copper efflux oxidase (CueO)^8^,^9^, fructose dehydrogenase (FDH)^10^,^11^,^12^, and PQQ-GDH^13^. However, the production of carbon gels is time-consuming, and controlling the amount of gel produced and its morphology with a satisfactory degree of reproducibility is difficult^14^. Mesoporous carbons can be prepared directly from carbon precursors through carbonization using hard templates (MgO, zeolites, mesoporous silicas, etc.) as well as soft templates (metal-organic frameworks and block copolymer surfactants, etc.)^15^,^16^,^17^,^18^. The use of mesoporous silica as a template, as first reported in 1999^19^,^20^. The pore size of the carbon material, as defined by the thickness of the silica wall of the mesoporous silica material, has a narrow distribution at around 5 nm; however, it is difficult to obtain a pore size reliably larger than the enzyme molecules. The most reliable synthetic route allowing control of morphology and pore structure for enzyme encapsulating is the hard template method using MgO^15^,^21^. Recently, a method for mesoporous carbon production using MgO as a template has been applied industrially^15^. MgO is a suitable template because it is both thermally and structurally stable during carbonisation, and is easy to remove from the resulting carbon by washing with a dilute acid. Another advantage of MgO-templated carbon (MgOC) over other mesoporous carbons is its tunable pore size distribution (2–150 nm), which can be modified by changing the crystalline structure of the template MgO. Furthermore, the interconnected mesopores of MgOC provide a high effective surface area per volume for enzyme immobilisation.

We have previously reported the DET reaction of BOD using a MgOC with pore diameters of 38 nm. The DET catalytic current density per geometric surface area was found to be 4 mA/cm^2^ with an electrode rotation rate of 8000 rpm at pH 7, 25 °C, with O_2_ saturation^22^; this DET catalytic current density was higher than that of carbon gel and was improved by introducing macropores for smooth mass transfer using 200-nm MgO particles as a template^23^. We also reported the effects of pore size on the current production efficiency of DET for FDH^24^. When the pore size of MgOC was comparable to the molecular size of FDH, the enzyme was stabilised by the presence of surrounding carbon mesopores. However, the catalytic current was limited by the amount of enzyme adsorbed in the mesopores at the surface of the carbon particles. In contrast, a high current density was obtained when the MgOC pore size was much larger than the FDH since enough FDH could be adsorbed in the mesopores on the surface of, and inside, the MgOC particles. To successfully satisfy the requirements of both a large specific surface area and stable entrapment for enzyme loading and rapid mass transport of fuel, a meso-macroscopic hierarchically structured carbon electrode is required. If the pores are considerably larger than the enzyme molecules, leaching and low loading can occur; however, if the pores are small, enzyme penetration will be prevented and will be located only at the surface of carbon. For constructing hierarchically structured carbon electrodes, two strategies could be considered. In one strategy, the macroporous structure is constructed first and the mesopores are formed on the surface of the macroporous material^25^,^26^, whereas in the other strategy, a mixture of porous carbon materials having different pore sizes is constructed on the substrate. There have been very few reports on meso-macroporous carbons worth mentioning. A three-dimensionally interconnected meso-macro bimodal porous silica sol–gel approach has been developed using a solution of polystyrene sulfonate spheres as the macro-template and tetraethyl orthosilicate as the silica particle precursor for mesopores, followed by multimodal porous carbon fabrication through the inverse replication of the bimodal silica^27^. The carbon has 6 nm mesopores and 200 nm macropores. MgO-templated carbon having bimodal mesopore size distribution (with maxima at 2 and 10 nm) were prepared using two MgO precursors, Mg citrate and Mg gluconate, with poly(vinyl alcohol) as the carbon precursor^28^. Compared to the size of the enzyme molecules, the mesopores in these materials are too small. To date, however, there has been no report concerning bimodal carbon material having mesopores larger than 20 nm and macropores.

In this study, we aimed to evaluate the effects of pore size on the catalytic current production efficiency and stability of BOD on an electrode using the MgOCs with different pore sizes imprinted from MgOs with crystal size of 40 nm for mesopores and 150 nm for macropores at different meso-macro ratios (MgOC_16_, MgOC_25_, MgOC_33_, MgOC_50_, MgOC_67_, MgOC_75_, MgOC_83_, the subscript number indicating the ratio of the 150-nm MgO template to the sum of the 40- and 150-nm MgO templates). The concept of this study is illustrated in Fig. 1.

## Results and Discussion

### Pore structure and morphology of MgOC

**Figure 1:**
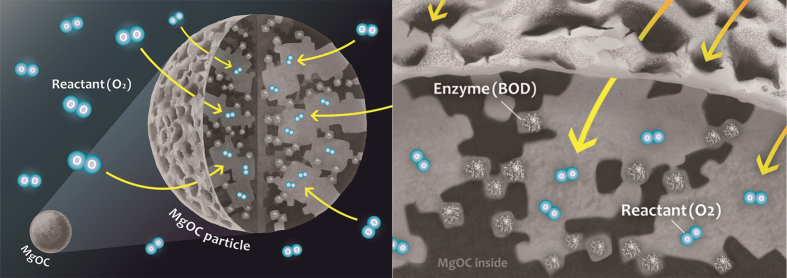
Schematic illustration of bimodal MgOC for electrochemical oxygen reduction reaction catalysed by bilirubin oxidase.

**Figure 2:**
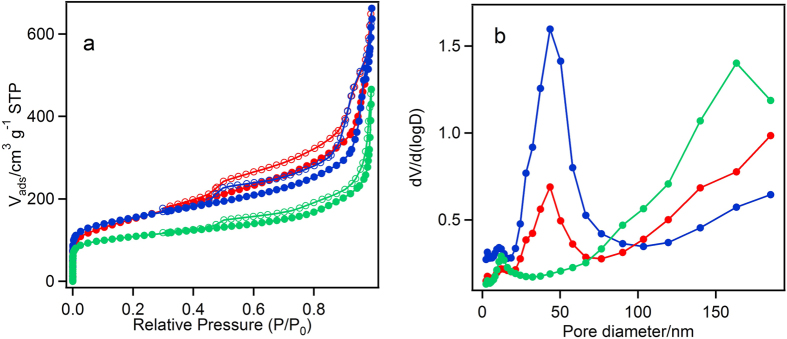
(**a**) N_2_ adsorption (closed circle) and desorption (open circle) isotherms and (**b**) the pore size distribution of MgOCs analysed by the BJH method. MgOC_meso_ (blue), MgOC_macro_ (green), MgOC_50_ (red).

**Figure 3:**
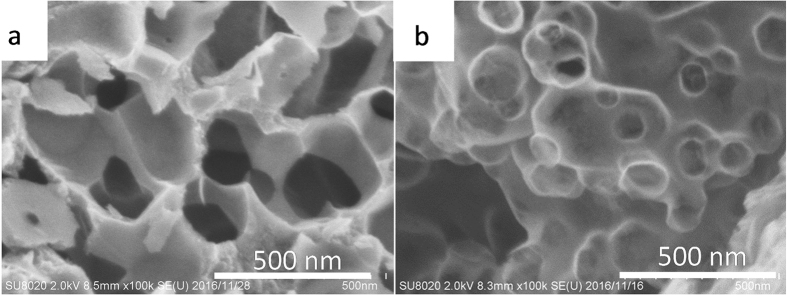
FE-SEM images of (a) MgOC_macro_ and (b) MgOC_50_.

The N_2_ adsorption and desorption isotherms of MgOC_meso_, MgOC_macro_, and MgOC_50_ and their pore size distribution, calculated using the BJH method, are shown in Fig. 2 and Table 1. The isotherms of the MgOCs were assigned as Type II with an H3-type hysteresis loop in the relative pressure (P/P_0_) between 0.8–1.0, which does not show a plateau in the region close to P/P_0_ = 1.0, and an H2-type hysteresis loop in the range P/P_0_ = 0.2–0.8. The H3-type hysteresis loop indicates the presence of slit–type mesopores, as well as macropores^29^,^30^. In contrast, the H2 hysteresis loop in the P/P_0_ range of 0.2–0.8, caused by the condensation in the capillary, indicates the presence of mesopores on the wall of the carbon macropores^31^,^32^. Although other hierarchical meso-macro-structured materials show similar N_2_ adsorption and desorption isotherms to those reported here, ours is the first report of a carbon material containing such a hierarchical structure^33^,^34^. The pore size distribution calculated using the BJH method shows characteristic curves depending on the materials, as shown in Fig. 2(b). Figure 3 shows FE-SEM micrographs of the surface of MgOC_macro_ (a) and MgOC_50_ (b). The MgOC_macro_ was derived from templating 150 nm of MgO crystalline. The image clearly reveals that the interconnected pores in the carbon are exactly replicating the morphology and the size of MgO crystals and suggests that MgO template formed by the pyrolysis of their precursors can leave mesopores with almost the same size and morphology in the resultant carbon. The image of MgOC_50_ (b) having bimodal pore size distributions (as shown in Fig. 2) shows interconnected macropores of 150 nm in width with mesopores of about 40 nm. The MgOC_50_, the carbon has the macropores were created by 150 nm of MgO templating and the mesopores with the size around 40–50 nm by 40 nm of MgO templating. Such branching pore structure was not observed in MgOC_meso_ and MgOC_macro_. Considering the SEM image and the pore size distribution analysed from N_2_ adsorption and desorption isotherms as shown in Fig. 2, MgOC adjusted by the dual template has a structure in which macropores and mesopores are hierarchically formed as shown in concept image as shown in Fig. 1.

### Effect of pore structure on the efficiency of O_2_ reduction current production

**Figure 4:**
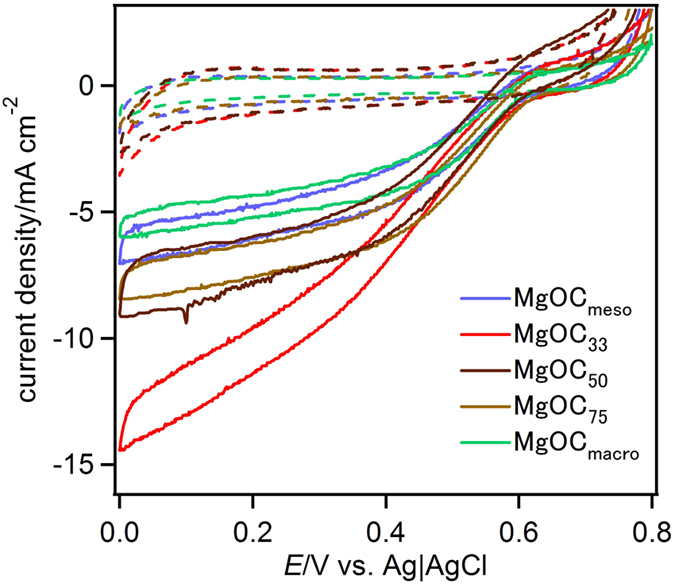
CV data for BOD adsorbed on a MgOC-modified electrode in a citrate buffer (0.1 M, pH 5.0) under O_2_-saturated conditions at 25 °C (solid curve). MgOC_meso_ (blue), MgOC_33_ (red), MgOC_50_ (blue), MgOC_75_ (purple), MgOC_macro_ (green). The scan rate was 1 mV s^−1^. Dashed curves indicate CVs without BOD.

Figure 4 shows the results of cyclic voltammetry (CV) experiments using MgOC electrodes that were rotated at 10,000 rpm as the working electrode in the presence (solid curve) and absence (dashed curve) of BOD in O_2_-saturated citrate buffer at pH 5.0 and 25 °C. The MgOC electrode was immersed in a BOD solution with stirring. The catalytic current density was calculated based on the geometric surface area of the electrode (0.071 cm^2^). The catalytic current in the CV (solid curve) indicates that BOD acts as an electrocatalyst for the reduction of O_2_ without an electron-transfer mediator. The onset potential of the catalysis, 0.57 V vs. Ag|AgCl, indicates that type 1 Cu (T1Cu) was the redox reactive site and that the reaction did not depend on the pore structure of MgOC^17^. The highest catalytic current density of the BOD-catalysed O_2_ reduction at 0.2 V was observed for MgOC_33_, while MgOC_macro_ and MgOC_meso_ had the lowest current density. The high current density seen for MgOC_33_ is thought to result from the uniform distribution of BOD in carbon particles containing 150 nm macropores. We suggest that macropores improve the mass transfer of BOD, leading to a high electroactive enzyme loading. The mesopores that form on the macropore walls accommodate BOD and exert a caging effect that improves the kinetics of heterogeneous electron transfer.

Among the bimodal MgOCs, the MgOC with a 2:1 ratio of meso- and macro-pores was found to be the optimal combination to allow both enzyme loading and mass transfer within the carbon particles. The results suggest that the pore structure and morphology, i.e., the macropore/mesopore distribution, affect the distribution of BOD in the carbon and leading to efficient current production. Unfortunately, the pore structure and morphology could not be determined by electron microscopy (FE-SEM, TEM, and STEM). Milling the samples with a focused ion beam followed by cross-sectional TEM imaging may allow the pore structure in the MgOC carbon particles to be determined in more detail.

The use of BOD-MgOC yielded the best O_2_ reduction current density and onset potential compared to other multicopper oxidases, including laccase and CueO, and BOD from other sources. The redox potential of BOD depends on the pH and is 0.53 V at pH 5^22^, which is very close to that of the T1Cu of fungal laccase (0.57 V vs. Ag|AgCl at pH 5.0)^11^. The onset potential in the O_2_ reduction voltammogram for BOD-catalysed O_2_ reduction is very close to that of laccase. Given the catalytic current production efficiency, CueO has a high affinity for the carbon surface and produces a relatively high current density even on a nonporous graphite surface. BOD can overcome the drawback in the low heterogenisous catalytic activity by the use of porous carbon to obtain a high enzyme loading with the proper orientation by caging the enzyme in the mesopores. An efficient current production of 27 mA cm^−2^ mg^−1^ could be realised with a lower enzyme and carbon loading (0.5 mg carbon per cm^2^ of GC surface) by selecting a suitable porous carbon material. The catalytic current density for BOD-catalysed oxygen reduction at an electrode modified with carbon nanotubes (CNTs) at pH 5.0 was ca. 4.6 mA cm^−2^ and the current density per unit weight of CNTs was 11.5 mA cm^−2^ mg^−1^ 35. The space within the nanopores could not be controlled in the CNT-modified electrode, and there may have been dead space that the enzyme could not enter. The dead space can be minimised by using pore-size-controlled porous carbon in which the pores are larger than the enzyme molecules. The efficiency of MgOC_33_ was 2.3 times greater than that of the CNT-based electrode, and the catalytic current density and current production efficiency of the MgOC_33_ are higher than any previously reported values for biocathodes.

**Figure 5:**
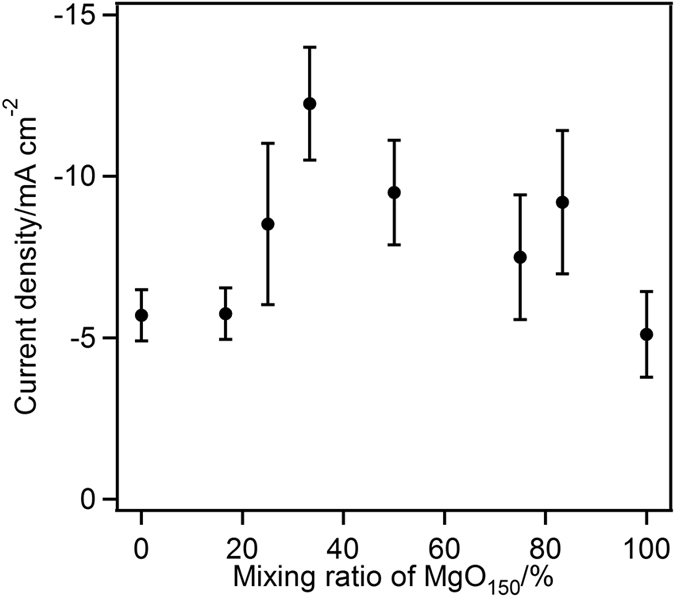
Dependence of the current density on pore morphology (macropore/mesopore ratio) at 25 °C during constant-potential electrolysis at 0.2 V.

Figure 5 shows the steady-state O_2_ reduction current density on the BOD-modified MgOC electrode observed during constant potential electrolysis at 0.2 V vs. Ag|AgCl for 120 s at an electrode rotation rate of 10,000 rpm. The BOD-modified MgOCs with mesopores and macropores produced catalytic current densities of −5.6 and −5.1 mA cm^−2^, respectively. The MgOCs containing both mesopores (40 nm) and macropores (150 nm) in different ratios show a complex relationship between the mesopore/macropore ratio and the catalytic current density. The highest current density was obtained for the carbon containing 33% macroporesquantity of macropores/(quantity of mesopores + macropores). The catalytic current density depends on the pore morphology, which is affected not only by the total amount of enzyme on the carbon but also by the mass transfer of O_2_ gas through the bulk solution to the adsorbed enzymes. Increasing the number of macropores (i.e. decreasing the number of mesopores) reduces the total surface area, thereby increasing the total amount of enzyme encapsulated in the mesopores. On the other hand, the bottle-neck structure formed by increasing the number of mesopores and decreasing the number of macropores (especially the macropores located on the surface of the carbon) prevents mass transfer of the O_2_ gas substrate within the carbon materials, thereby decreasing the efficiency of the utilization. The result suggests that a mixture of 33% macropores and 67% mesopores was optimal for the BOD-catalysed oxygen reduction cathode.

**Figure 6:**
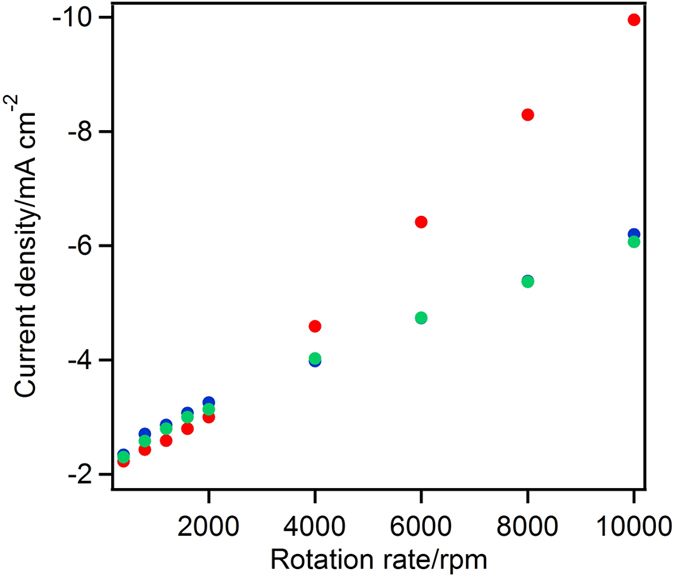
Rotation rate dependence of the current density. MgOC_macro_ (green), MgOC_33_ (red), and MgOC_meso_ (blue).

Figure 6 shows the rotation rate dependence of the O_2_ reduction catalytic current density for the MgOC_macro_-, MgOC_meso_-, and MgOC_33_-modified electrodes. The current densities of these three electrodes did not depend on the rotation rate below 2,000 rpm. This result suggests that the catalytic current depends on the mass transfer of O_2_ from the bulk solution to the electrode surface. Interestingly, when the electrode rotation rate was increased beyond 2,000 rpm, the current of the MgOC_33_ electrode showed a greater increase with increasing rotation rate than the MgOC_macro_ and MgOC_meso_ electrodes. This behaviour agrees with a previous report by Heller’s group, who used a carbon textile electrode modified with a redox hydrogel containing glucose oxidase^36^. The catalytic current depending on the rotation rate can be determined by the enzymatic reaction rate including the total amount of electrochemically active enzyme on the carbon surface, and mass transfer efficiency of O_2_. This result suggests that (1) the macropores allow more BOD to adsorb both on the carbon particle surface and inside the carbon particles, (2) the enzymes inside the carbon particle are surrounded by mesopores, which enables their high electrochemical activity, (3) further increase of the enzyme in the low-surface area macropores (MgOC_macro_) could prohibit the electrochemical reaction by the formation of adsorbed multilayers in the macropore, (4) O_2_ penetration from the bulk solution during electrochemical reactions is smoother through a MgOC carbon layer with macropores than through the carbons with only mesopores. (5) The mass transfer of O_2_ was restricted in MgOC_meso_, during electrochemical reactions. Furthermore, the inability of BOD to penetrate the solely mesoporous carbon particles (MgOC_meso_) leads to the formation of a BOD monolayer on the surface of the porous carbon particles during modification of the electrode with BOD.

### Effect of pore structure on the thermal and adsorption stability of adsorbed BOD

**Figure 7:**
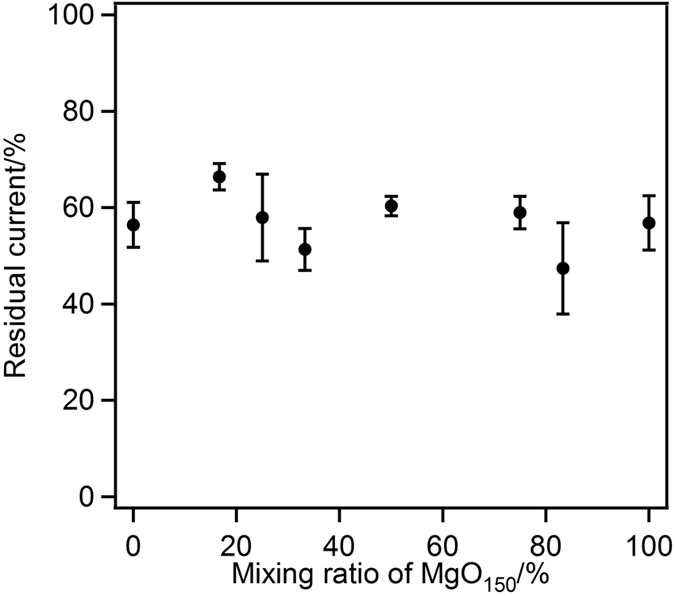
Dependence of the thermal stability of BOD-modified electrode after treatment at 50 °C on the pore morphology (macropore/mesopore ratio).

After the constant-potential electrolysis experiment at 25 °C (Fig. 5), the electrode was dipped in a citrate buffer solution (50 °C, pH 5.0) for 10 min. No potential was applied to the electrode during the heat treatment. The residual current density was determined as the ratio of the current densities obtained before and after the heat treatment. Figure 7 shows the dependence of the residual current density in the BOD-catalysed reduction of O_2_ on the macropore/mesopore structure (ratio of the number of macropores to the total number of pores). The current density of all MgOC electrodes decreased to almost 60% of the initial current density after heat treatment, and no difference was observed when the temperature was kept at 25 °C. The BOD caged in the mesoporous support was protected from the heat treatment; the mesopores may facilitate the retention of the 3D structure of BOD in the pores, and the desorption of BOD from the carbon surface might be limited. A similar stabilisation effect was reported for mesoporous carbon in our previous study using FDH^19^. In the previous report, the thermal stability of FDH in macropores was lower than that in mesopores. However, the stability of BOD did not depend on the pore size. BOD tended to adsorb in multilayers in the macropores, which causes BOD units to interact each other to increase their stability^37^,^38^.

**Figure 8:**
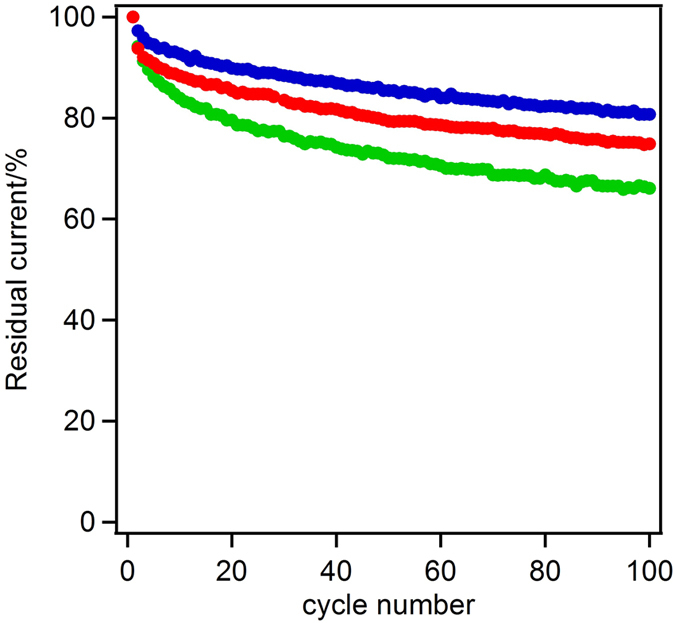
Stability of the electrode response during rotation at 10,000 rpm for the various pore morphologies (macropore/mesopore ratio) at 15 °C. MgOC_meso_ (blue), MgOC_macro_ (green), and MgOC_33_ (red).

Figure 8 shows the adsorption stability of the enzyme on the carbon electrode under while the electrode stirred vigorously by rotation at 10,000 rpm. The applied potential was cycled from 0.8 to 0 V at a scan rate of 10 mV s^−1^. The experiments were conducted at 15 °C to capture the effects of enzyme desorption without the interference from the thermal denaturation of the enzyme. The BOD-MgOC_meso_ electrode retained 80% of its initial current after 100 cycles, showing the highest residual current density under these conditions. The MgOC_macro_ electrode retained approximately 66% of its initial current density, and the macroporous/mesoporous carbon retained 75% of the initial current density. The curves showing the decrease in current density indicate that the MgOCs undergo a two-step degradation process; first, a rapid drop in activity and then a gradual decrease was seen. The first process is thought to be related to desorption of the enzyme from the pores on the surface of carbon particles. The second, slower degradation process involves enzyme desorption from the pores inside the carbon particles. There was no difference between the slopes of the curves of the MgOCs with mesopores and those of the MgOCs with both meso- and macropores. The enzymes caged in the mesopores show a high adsorption stability, and this result also supports the findings of the thermal stability test (Fig. 7).

The results shown in Fig. 5 suggest that the presence of macropores can increase the mass transfer rate in the carbon, thereby providing a large effective surface for the electro-enzymatic reaction. The results shown in Fig. 7 imply that the enzyme is stabilised by immobilisation in mesopores. A porous carbon-containing a mixture of macropores and mesopores consisting of 33% of mesopores exhibited both high electroactive enzyme loading and increased mass transfer. The stabilisation of BOD from *Myrothecium verrucaria* (Amano Enzyme, Japan) by its immobilisation in a silica sol–gel/CNT composite electrode has been previously reported^39^. The residual activity after heat treatment for 10 min at 49 °C for the silica-CNT system was approximately 50%, and the catalytic current density was 0.15 mA cm^−2^. The porous carbon electrodes reported herein exhibited a catalytic current more than 60 times higher than the silica-CNT system and achieved greater enzyme stability.

## Conclusions

BFC construction is simplified by the use of DET electrodes because there is no need for diffusional redox mediators or an ion-conducting separator. The removal of these inhibitory requirements enables the design of membrane-free BFCs, opening the possibility of further miniaturisation. The low stability and low current production efficiency of DET electrodes compared to those of MET systems would be insurmountable barriers to their application in BFCs. The use of a porous carbon material with a controlled pore structure and morphology allows simultaneous improvement of the stability and current density. In this paper, we report the effect of pore size and morphology on the current production efficiency of an enzyme-based bioelectrocatalyst. Based on these results, we suggest a new strategy for designing porous carbon materials with a controlled macropore/mesopore morphology by mixing templates of different sizes. Macropore increase the mass transfer of biocatalyst and fuel, gas, and electrolyte. Mesopore can increase the current production efficiency by encaging the enzyme, reducing the electron transfer distance. For improving the heterogeneous electron transfer rate further, the process reported here must be combined with the designing of nanostructures (nanointerface), and chemical properties must be tuned by introducing specific molecules and hetero-atom doping^40^. The process reported here can be applied to other redox enzyme systems, including DET- and MET-type anodes and cathodes. Tailor-made porous carbon will unlock a new era in the fabrication and application of enzyme electrodes.

## Methods

### Enzymes and reagents

BOD was purchased from Amano Enzyme (Japan) and used without further purification. The concentration of the BOD stock solutions was spectrophotometrically determined using the molar extinction coefficient of BOD at 600 nm (4,800 M^−1^ cm^−1^)^41^. The MgOCs with different pore sizes used in this study, MgOC_meso_, MgOC_16_, MgOC_25_, MgOC_33_, MgOC_50_, MgOC_67_, MgOC_75_, MgOC_83_, and MgOC_macro_, were kindly donated by Toyo Tanso (Japan)^15^,^16^. MgOC_meso_ and MgOC_macro_ were prepared from MgO with crystal sizes of 40 and 150 nm, respectively. “MgOC” with a subscript number indicates that the MgOC contains both macro- and mesopores; the number indicates the ratio of the 150 nm MgO template to the sum of the 40 and 150 nm templates.

### Characterization of the pore structure and morphology of MgOCs

The carbon structures were examined by field emission scanning electron microscopy (FE-SEM, SU-8020, Hitachi, Japan). The pore size distributions and specific surface areas of the MgOCs were evaluated from N_2_ adsorption and desorption isotherms obtained at 77 K using an adsorption apparatus (BELSORP MAX, Japan). Before the adsorption isotherm measurements, the MgOCs were heated at 300 °C for 3 h in a vacuum to clean their surfaces. The Brunauer–Emmett–Teller (BET) surface area (*S*_BET_), average pore diameter (*d*_p_), micropore volume (*V*_micro_), and total pore volume (*V*_total_) of the MgOCs were evaluated from the N_2_ adsorption isotherms. The *d*_p_ and *V*_micro_ values were obtained by applying the Barrett–Joyner–Halenda (BJH) method. *V*_total_ was calculated from the amount of nitrogen adsorbed at a relative pressure (P/P_0_) of 0.997.

### Fabrication of BOD modified MgOC electrode

The poly(vinylidene difluoride) binder (200 μL, 5% in *N*-methyl pyrrolidone (NMP), Kureha, Japan) and MgOC particles (40 mg) were mixed in NMP (0.1 mL) using a tip-type ultrasonicator (SMT UH-50, Japan) for 2 min. Carbon ink (4 μL) was applied to a glassy carbon electrode (GCE, 3 mm in diameter, BAS, Japan). The electrode was dried in an oven at 60 °C for 12 h to obtain the MgOC-modified electrode. The electrode was dipped in ethanol for 1 min and then rinsed with a citrate buffer solution (pH 5). The MgOC-GCE was immersed in a BOD solution (1.7 μM in a solution of potassium phosphate solution at pH 8.5) with stirring for 6 h at 4 °C and used as the working electrode.

### Electrochemical measurements

The BOD-immobilized MgOC-GCE was used as the working electrode. Electrochemical measurements were performed using an electrochemical analyser (BAS CV 50 W, BAS Inc.) with a platinum wire and an Ag|AgCl|KCl(sat.) electrode as the counter and reference electrodes, respectively. The working electrode was rotated using an RDE-2 instrument (BAS, Japan). All electrochemical measurements were performed in a water-jacketed electrochemical cell in a citrate buffer (100 mM, pH 5). The temperature of the electrolyte slution was maintained at 25 °C by an isothermal circulator unless otherwise specified.

## Additional Information

**How to cite this article:** Funabashi, H. *et al*. Hierarchical meso/macro-porous carbon fabricated from dual MgO templates for direct electron transfer enzymatic electrodes. *Sci. Rep.* **7**, 45147; doi: 10.1038/srep45147 (2017).

**Publisher's note:** Springer Nature remains neutral with regard to jurisdictional claims in published maps and institutional affiliations.

## Figures and Tables

**Table 1:** Pore properties of MgOCs, as calculated from N_2_ adsorption-desorption isotherms and BJH analysis.

	Specific surface area (m^2^/g)	Total pore volume (mL/g)	Micro pore volume (mL/g)
MgOC_meso_	590	0.67	0.23
MgOC_macro_	420	0.39	0.17
MgOC_16_	570	0.55	0.22
MgOC_25_	570	0.56	0.22
MgOC_33_	580	0.54	0.23
MgOC_50_	580	0.60	0.23
MgOC_75_	500	0.56	0.21
MgOC_83_	510	0.54	0.21
